# DIABETES MELLITUS AND HARD BRAKING EVENTS IN OLDER DRIVERS

**DOI:** 10.1093/geroni/igac059.2738

**Published:** 2022-12-20

**Authors:** Difei Liu, Stanford Chihuri, Thelma Mielenz, Linda Hill, Carolyn DiGuiseppi, David Strogatz, Guohua Li

**Affiliations:** Columbia University Medical Center, New York, New York, United States; Columbia University, New York, New York, United States; Columbia University, New York, New York, United States; University of California San Diego, La Jolla, California, United States; University of Colorado Anschutz Medical Campus, Aurora, Colorado, United States; Bassett Research Institute, Cooperstown, New York, United States; Bassett Research Institute, Cooperstown, New York, United States

## Abstract

There are an estimated 37 million people with diabetes mellitus (DM) in the United States, including 16 million older adults. DM can impair patients’ driving safety due to diabetic peripheral neuropathy, hypoglycemia, or hyperglycemia, and eye diseases. However, few studies have examined the association between DM and driving safety based on naturalistic driving data. Data for this study came from the Longitudinal Research on Aging Drivers (LongROAD) project, a multisite naturalistic driving study of 2990 drivers aged 65–79 years at baseline. Driving data for the study participants were recorded by in-vehicle recording devices for up to 44 months. We used multivariable negative binomial modeling to estimate the incidence rate ratios (IRRs) and 95% confidence intervals (CIs) of hard braking events (i.e., proxies for unsafe driving behavior defined as maneuvers with deceleration rates ≥ 0.4 g) associated with DM. Of the 2856 study participants eligible for this study, 482 (16.9%) reported having DM at baseline. The overall incidence rate of hard braking events was 1.16 per 1000 miles. Adjusted for age, race/ethnicity, marital status, education level, annual household income, urbanicity, history of stroke, and number of medications, drivers with DM had a 10% increased rate of hard braking events compared to drivers without DM (adjusted IRR 1.10; 95% CI: 1.08, 1.12). Results of this study indicates that DM is associated with a significantly increased rate of hard braking events in older drivers, suggesting less safe driving. Driving safety should be incorporated into DM management and care programs.

